# Near-Infrared Spectroscopy and Multivariate Analysis as an Effective Method to Discriminate *Escherichia coli* in Clinical Samples

**DOI:** 10.1021/acsomega.5c04368

**Published:** 2025-06-23

**Authors:** Lavínia H. S. Pereira, Ayrton L. F. Nascimento, Larissa E. Mesquita, Renato M. Neto, Kássio M. G. de Lima

**Affiliations:** † Universidade Federal do Rio Grande do Norte, Instituto de Química, Programade Pós-Graduação em Química, Laboratório de Química Biológica e Quimiometria, Natal, Rio Grande do Norte CEP 59072-970, Brazil; ‡ Laboratório de Micobactérias - Departamento de Microbiologia e Parasitologia, UFRN, Natal 59072-970, Brazil

## Abstract

*Escherichia coli* is a bacterium that inhabits the gastrointestinal system and is considered to be an essential part of the intestinal microbiota. However, some strains can be pathogenic, causing urinary tract infections. These bacteria can develop antibiotic resistance during prolonged or inadequate treatments, and sensitive and specific tests are necessary for diagnosis. In this study, we aimed to determine, through NIR spectroscopy combined with variable selection techniques such as the successive projections algorithm (SPA) and genetic algorithm (GA) integrated with linear discriminant analysis (LDA), the discrimination of *E. coli* strains (sensitive vs resistant). The two *E. coli* strains resulted in a total of 162 spectral data, classified into 81 sensitive and 81 resistant spectra. These data were later subdivided into 114 for training, 24 for validation, and 24 for testing. Each of these sets maintained a balanced proportion between the two strains, containing half of the sensitive and half of the resistant strains. The variables selected by these methods were used to differentiate the species. Additionally, we evaluated the influence of spectral preprocessing techniques such as Savitzky–Golay smoothing and extended multiplicative scatter correction (EMSC) on the spectral data. The results showed that both models (SPA-LDA and GA-LDA) presented 100% sensitivity and specificity for both sensitive and resistant strains. This demonstrates that NIR spectroscopy combined with variable selection techniques can be an effective method for rapid and accurate identification of bacterial strains, offering a promising alternative for microbiological diagnostics.

## Graphical abstract

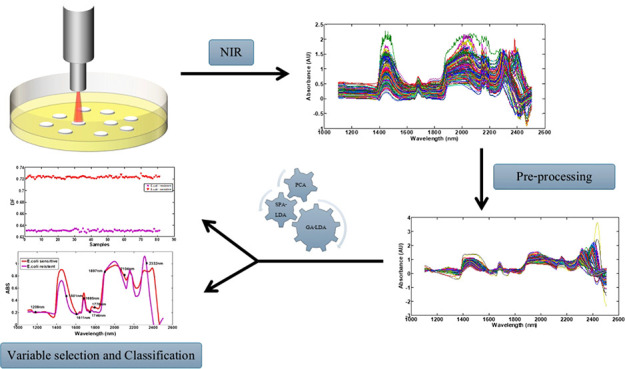

## 1 Introduction

Infectious diseases have been gradually increasing worldwide and are responsible for major morbidities and mortalities across the globe due to the rise of bacteria that are increasingly resistant to antibiotics.^1,2^ The emergence and spread of bacteria resistant to various types of drugs have hindered the treatment of common infections.^3^ For example, isolates of *Escherichia coli*—commonly known as *E. coli*—constitute a group of bacteria that reside in the human intestine and typically present a normal colonization density between 10^7^ and 10^8^ colony-forming units (CFU).^4^ These bacteria are responsible for causing urinary tract infections and increasing the number of hospital-acquired infections, which are becoming increasingly difficult to treat due to the resistance of *E. coli* to some types of antibiotics.^5^ The extended-spectrum beta-lactamase (ESBL) strains of *E. coli* are Gram-negative and play an important role in resistance to various classes of antibiotics, representing a major challenge in treating these infections.^6^ ESBLs confer resistance to beta-lactam antibiotics, resulting in limited treatment options for these bacterial infections.^5^ The group of β-lactamase enzymes, identified as extended-spectrum β-lactamases (ESBL), is composed of several Gram-negative bacteria and is responsible for the hydrolysis-based inactivation of β-lactam antibiotics.^7^

Traditional methods used to identify microorganisms, such as bacteria, typically require an estimated 2–5 days or more, including physiological, morphological, chemical, and biochemical characterization. Moreover, most phenotyping methods for microbial identification are time-consuming, labor-intensive, costly, and require substantial material.^8^ However, advanced spectroscopic techniques such as ATR fluorescence, FTIR, and EEM have been applied to identify cancer cells,^9,10^ and Raman spectroscopy has been used to detect phospholipids and proteins in blood. Spectroscopic techniques do not require specific reagents and allow for nondestructive and noninvasive measurements,^9^ offering rapid results and demonstrating robustness, high sensitivity, and versatility, making them widely explored as diagnostic tools. Vibrational spectroscopy—particularly infrared (IR) and Raman techniques—has shown promise in bacterial diagnostics, especially in strain typing and in distinguishing Gram-positive from Gram-negative samples.^11^

A viable and alternative approach is the application of near-infrared spectroscopy (NIRS), which enables the detection of analyte samples and has proven satisfactory in identifying microbial species.^12^ NIR is sensitive in the absorption regions of CH, NH, and OH groups, which relate to the microbial components. Additionally, it provides easy sample preparation, rapid response time, is nondestructive, and offers low instrumentation costs compared to other spectroscopic techniques such as ultraviolet, visible, mid-infrared (MIR), and Raman.^12^ This spectroscopic technique is widely used in analytical chemistry and is gaining prominence in microbiology for studying the bacterial cell membrane structure and the presence of lipids, proteins, and polysaccharides in these species.^13^ Various developments and advances have contributed to the successful application of NIR spectroscopy in microbial species. The use of multivariate analysis associated with NIR spectroscopy enables the extraction of qualitative and quantitative information from complex spectra for bacterial characterization—essential for the advancement of NIR technology. Principal component analysis (PCA), for example, aims to reduce data dimensionality by projecting it onto dominant components or scores, retaining relevant variance within the data.^14^ Identification through spectral similarity between microorganisms is performed via hierarchical cluster analysis (HCA),^15^ and for sample classification across different classes, linear discriminant analysis (LDA)^16^ is applied to group similar samples and distinguish differing ones. LDA, when combined with dimensionality-reduction algorithms such as PCA, successive projections algorithm (SPA), and genetic algorithm (GA), improves model performance compared to using the full spectrum.^16,17^

Several studies have shown promising results using NIR spectroscopy combined with variable selection techniques to distinguish bacterial species, especially *Pseudomonas aeruginosa*. This approach enabled the differentiation between sensitive and resistant strains. Among the evaluated algorithms, SPA-LDA and GA-LDA performed the best, with GA-LDA showing superior results. The wavelengths selected by the latter model highlighted bands associated with C–H groups (indicative of lipids) and overtones related to OH bond stretching, which are distinctive features of the analyzed strain.^17^ In addition to distinguishing *P. aeruginosa*, the technique was successfully employed to identify *Escherichia coli* and *Salmonella enteritidis* strains in pineapple juice pulp. The models applied—PCA, SIMCA (soft independent modeling of class analogy), and PLS-DA (partial least squares discriminant analysis)—showed promising performance in differentiating between the two species and detecting their presence in the fruit matrix.^14^

The increasing incidence of infections caused by microorganisms, such as *Escherichia coli*, represents a serious global public health issue. The application of conventional methods is time-consuming and expensive. Therefore, this work is justified by the need for innovative diagnostic methods that help curb antimicrobial resistance while promoting speed and efficiency in clinical settings. In this context, this study aims to apply NIR spectroscopy combined with variable selection techniques to identify *E. coli* strains isolated from clinical material—both sensitive and multidrug-resistant. We employed SPA and GA to select a suitable subset of wavelengths for LDA to characterize differences between strains, offering an effective, reliable, and low-cost method for the rapid identification of these microorganisms.

## 2 Material and Methods

### 2.1 Bacteria Strains

Two bacterial strains of the species *Escherichia coli* were used in this study: the standard ATCC 25922 strain and a strain with phenotypic and genotypic profiles characteristic of extended-spectrum beta-lactamase (ESBL), both isolated from biological material provided by the culture collection of the Mycobacteria Laboratory (Labmic) of the Federal University of Rio Grande do Norte (UFRN), Natal/RN, Brazil. The study was approved by the UFRN Research Ethics Committee under decision no. 331/2012.

### 2.2 Maintenance of Strains

The isolates confirmed to be ESBL producers were subjected to molecular identification of the genes encoding extended-spectrum beta-lactamases CTX-M, SHV, and TEM using the polymerase chain reaction (PCR) technique. After the etiological and phenotypic resistance profiles were confirmed, the strains were subjected to successive subcultures in new growth medium to ensure the reproducibility of the results. Subculturing of both strains was carried out on 42 nutrient agar plates, with 14 plates for the sensitive strain and 28 plates for the resistant strain. Following this, studies of each colony were initiated. After the study, the strains were removed from the culture collection of the Mycobacteria Laboratory—Labmic, Federal University of Rio Grande do Norte, RN, Brazil. In this study, the spectral data were organized into two classes: Class 1, composed of 81 spectra corresponding to samples with a phenotype sensitive to antibacterial agents; and Class 2, also composed of 81 spectra, corresponding to samples confirmed as multidrug-resistant to beta-lactam antibiotics.

### 2.3 NIR Spectroscopy

Bacterial species were transferred from the stock study to nutrient agar plates (HIMEDIA) and maintained in a bacteriological incubator at 35 °C. Each NIR spectrum (spectral resolution of 8 cm^–1^) was acquired directly in reflectance mode, where the detector was adjusted for triplicate analyses (isolated colonies) to obtain maximum variability within the same sample and between different samples, using a miniature Fourier-transform scanning spectrometer (ARCspectro ANIR, Neuchâtel, Switzerland). The portable NIR device uses an InGaAs photodiode (900–2600 nm), and the reflected light was directed to the spectrometer via a fiber-optic bundle (model R600-7-VIS-125F, Ocean Optics, USA) connected to the probe tip. Data acquisition and analysis were carried out using ARCspectro ANIR 1.64 software.

### 2.4 Multivariate Analysis

The import of data, preprocessing, and construction of chemometric classification models (SPA-LDA and GA-LDA) were implemented in MATLAB R2014a software (MathWorks Inc., Natick, MA, USA). The NIR spectra were preprocessed using the Savitzky–Golay filter with a 15-point window, second-order polynomial, and zeroth derivative, and using extended multiplicative scatter correction (EMSC) with a second-order polynomial. Mean centering was applied to all spectra before variable subset selection and calibration.

For the SPA-LDA and GA-LDA models, samples were divided into training, validation, and test sets using the classical Kennard–Stone (KS) uniform sampling algorithm on the NIR spectra.^15^ The number of samples in each set is listed in Table 1. The training samples were used for the modeling procedure (including variable selection for LDA and QDA), while the prediction set was used solely for final classification evaluation. The optimal number of variables for SPA-LDA and GA-LDA was determined from the minimum cost function *G*, calculated for a given validation data set:

1 $$G=\frac{1}{N_{v}}\overset{N_{v}}{\underset{n=1}{\sum }}g_{n}$$

**Table 1:** Number of Training, Validation, and Test Spectra in Each Category

Category	Training	Validation	Test
(1) *E. coli* sensitive	57	12	12
(2) *E. coli* resistant	57	12	12
Total	114	24	24

where *N*_v_ refers to the number of validation samples, and *g*_*n*_ is defined as

2 $$g_{n}=\frac{r^{2}\left(x_{n},m_{I\left(n\right)}\right)}{\mathrm{mi}n_{I\left(m\right)I\neq \left(n\right)}r^{2}\left(x_{n},m_{I\left(n\right)}\right)}$$

a discriminant metric or value for sample *n*, used to identify the class to which *x_n_*, belongs. The numerator *r* ^2^(*x_n_*,* m*_*I*(*n*)_) in eq 2 corresponds to the squared distance between point *x_n_*, and the centroid of the class to which it belongs. The denominator min_*I*(*m*)*I*≠(_ *_n_*_)_ *r* ^2^(*x*_*n*_,*m_I_*_(*n*)_) in eq 2 refers to the shortest squared distance between *x*_*n*_ and the centroids of the other classes, excluding *I*_(*n*)_. The GA-LDA was applied with an initial population of 2 individuals across 100 generations. Crossover and mutation probabilities were set at 10% and 60%, respectively, and the process was repeated three times, starting from different random initial populations.

In this study, precision measures such as sensitivity (the probability that the test result is positive when the disease is present) and specificity (the probability that the test result is negative when the disease is absent) were used to evaluate test performance. Both measures range from 0 to 1.^16^

$$\mathrm{Sensibility}\;\left(\%\right)=\frac{\mathrm{TP}}{\mathrm{TP}+\mathrm{FN}}\times 100$$

$$\mathrm{Specificity}\;\left(\%\right)=\frac{\mathrm{TN}}{\mathrm{TN}+\mathrm{FP}}\times 100$$

where FN is defined as false negative, FP as false positive, TP as true positive, and TN as true negative.

## 3 Results and Discussion

In this study, differentiating between *E. coli* ATCC and *E. coli* ESBL strains represents a significant clinical challenge due to the close phylogenetic proximity between them. This similarity complicates conventional diagnosis, requiring more precise methods to correctly identify strains and distinguish their specific characteristics.

Figure 1A shows the near-infrared (NIR) spectra in the 1000–2600 nm range of bacterial suspensions from the two *E. coli* strains (sensitive vs resistant). It was difficult to distinguish differences in the raw NIR spectra between the classes due to the high degree of band overlap.

**Figure 1:**
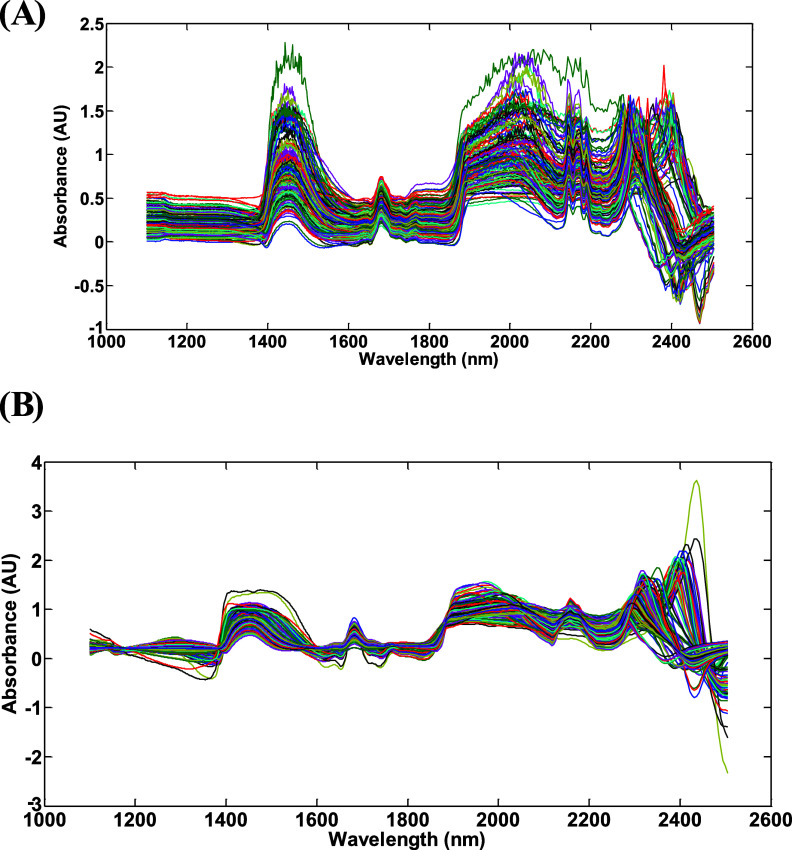
Original near-infrared spectra of bacterial suspension from *E. coli* (A), NIR spectra with first derivative of the Savitzky–Golay using a window of 15 points, and by extended multiplicative scattering correction (EMSC), with polynomial 2 (B).

In this context, some preprocessing techniques were applied (Figure 1B). The first preprocessing step used extended multiplicative scatter correction (EMSC) with a second-order polynomial to normalize and remove the baseline. This model is defined based on a reference spectrum, making the EMSC modeling very stable—even when spectral changes are due to sample thickness variations.^19^ Subsequently, the second derivative (Savitzky–Golay) was applied to the spectra. This derivative transformation is commonly used to process spectral data by separating overlapping absorption bands, removing baseline shifts, and enhancing apparent spectral resolution.^14^

### 3.1 PCA

When PCA was applied to the NIR data, two distinct groups corresponding to each class (sensitive—ATCC vs resistant—ESBL) were formed, showing a two-dimensional distribution of the samples in relation to the principal components PC1 and PC2 (Figure 2). The plot demonstrates the separation between the sensitive (ATCC) and resistant (ESBL) strains, highlighting a variance of 95%. A clear distinction is observed along the PC1 axis, indicating that this principal component retains the most variability associated with the ESBL resistance profile. Despite the separation, PCA alone did not achieve satisfactory class distinction, as there is a region of overlap between the groups. This suggests the presence of shared phenotypic or molecular characteristics among the samples. Additionally, the dashed blue ellipse represents the 95% confidence interval, reinforcing that most samples fall within the expected range of variation. The scattered distribution of some samples outside the confidence ellipse suggests the presence of possible outliers or atypical samples. These may be linked to intrinsic variability among ATCC and ESBL strains or experimental factors such as spectral noise or unique chemical profiles. The observed dispersion in ATCC samples may indicate greater heterogeneity compared to the resistant isolates, which showed more homogeneous clustering, suggesting a more uniform resistance profile. Given this variability, it becomes necessary to apply mathematical methods—such as variable selection techniques—to enhance model performance and enable reliable application to unknown samples, thereby contributing to more accurate future clinical diagnoses.

**Figure 2:**
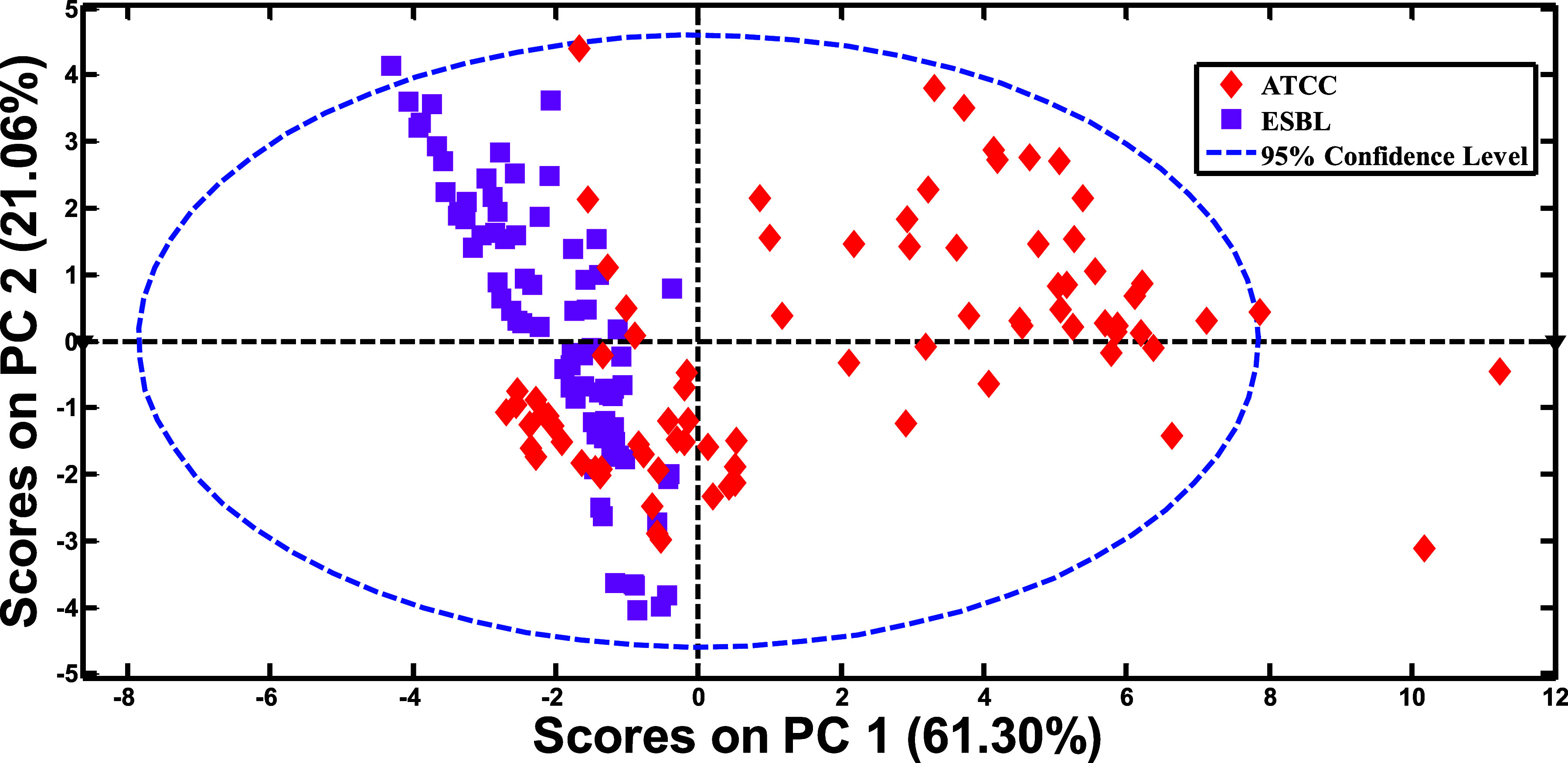
PCA scores for *E. coli* ATCC and *E. coli* ESBL.

### 3.2 SPA-LDA and GA-LDA Models

In this study, variable selection techniques—successive projections algorithm (SPA) and genetic algorithm (GA)—combined with linear discriminant analysis (LDA) were applied to the spectral data with two main objectives. The first was to develop a predictive model capable of discriminating unknown *Escherichia coli* strains (sensitive and resistant), assessing the test’s accuracy through sensitivity and specificity results. The second objective was to identify alterations in the biochemical “fingerprint” of the strains based on the variables selected by each combined approach. The relevant wavelengths extracted by both the SPA-LDA and GA-LDA models are shown in Table 2.

**Table 2:** Selected Wavelengths by SPA-LDA and GA-LDA, respectively

SPA-LDA Selected Wavelengths	Assignment
1401 nm	First overtone of alcohols (−OH)
2148 nm	Combination overtone of primary amides
2294 nm	C=O bond with N–H group in peptide structures
2319 nm	Presence of methylene C–H
2354 nm	C–H bond in polysaccharides
2372 nm	Second overtone of alcohol (−OH) bond
2395 nm	Presence of aromatic C–H bond
2427 nm	–
2451 nm	C=O bond with N–H group in peptide structures
2485 nm	C–H stretching and combination of C–C stretching (cellulose)

GA-LDA Selected Wavelengths	Assignment
1209 nm	C–H (CH_2_) methylene bond
1501 nm	First overtone of alcohol (−OH) bond
1611 nm	Combination overtone of primary amides (CONH_2_)
1685 nm	First overtone of aromatic C–H bond
1745 nm	First overtone of C–H (CH_2_) methylene bond
1776 nm	C–H (CH_2_) methylene bond
1897 nm	Second overtone of carboxylic acid (−COOH)
2104 nm	COO bond in polysaccharides

The application of SPA combined with LDA allowed the selection of wavelengths with high discriminative power between sensitive and resistant *E. coli* strains. Figure 3A shows the average absorbance (ABS) spectra of both strains in the NIR region. The spectral profile differences reveal biochemical changes associated with the resistance phenotype. Eleven relevant wavelengths were selected (Table 2), indicating structural and metabolic variations between the two strains. Some wavelengths selected by SPA-LDA stood out, notably 2395, 2372, 2148, and 1401 nm, corresponding respectively to aromatic C–H bonds, the second overtone of the O–H alcohol bond, the combination overtone of primary amides, and the first overtone of alcohols.^18,20^ For sensitive *E. coli* strains (Class 1), both sensitivity and specificity reached 100%. Similarly, the resistant *E. coli* strains (Class 2) also achieved 100% sensitivity and specificity, demonstrating the model’s effectiveness in identifying spectral patterns related to bacterial resistance.

**Figure 3:**
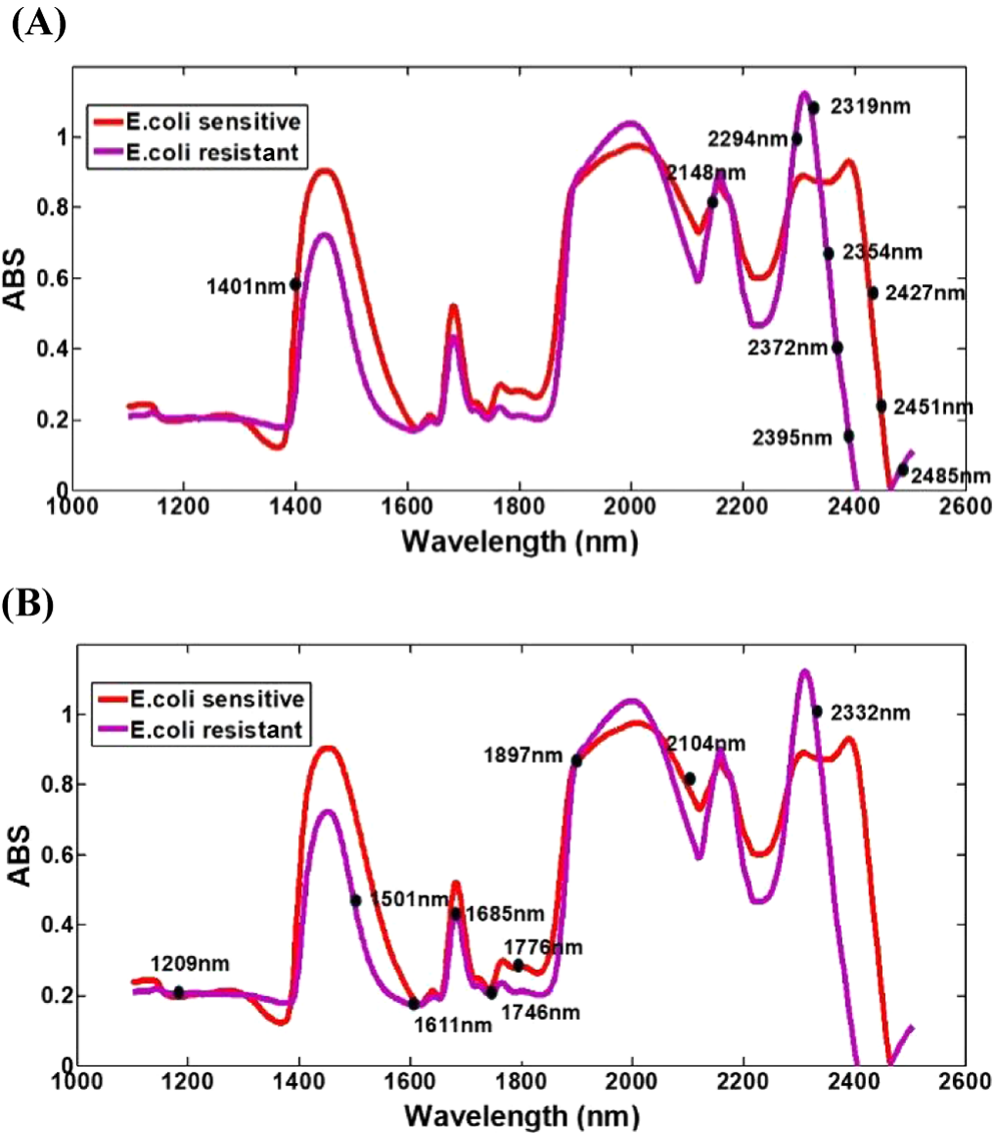
SPA-LDA (A) and GA-LDA (B) selected variables on preprocessed mean spectra for *E. coli*ATCC (sensitive) in red and *E. coli* ESBL (resistant) in purple.

The GA-LDA model identified nine specific wavelengths (Table 2) with high discriminative power between sensitive and resistant *E. coli* strains. The highlighted spectral peaks in Figure 3B reveal regions that reflect absorbance changes potentially related to structural and biochemical modifications characteristic of resistant bacteria. The model achieved 100% sensitivity and specificity. Among the wavelengths selected by GA-LDA, notable ones include 1685, 1611, 1897, and 1501 nm, corresponding respectively to the first overtone of aromatic C–H bonds, the combination overtone of primary amides (−CONH_2_), the second overtone of carboxylic acid (−COOH), and the first overtone of alcohol (−OH).^18,20^ The GA operates as a stochastic algorithm inspired by natural selection, optimizing the selection of the most relevant variables within the spectral data set. LDA then evaluates the ability of these variables to maximize separation between predefined groups. This combined approach is particularly advantageous in NIR spectroscopy, where data are typically highly collinear and noisy, requiring robust techniques to avoid overfitting and ensure good generalization. Thus, the selected wavelengths not only simplify the classification model but also provide a basis for developing portable and efficient optical sensors capable of rapidly distinguishing bacterial strains with different resistance profiles—without the need for time-consuming traditional microbiological methods.

Figure 4A highlights the discriminant scores generated by the SPA-LDA model, showing its effectiveness in separating the two *E. coli* strains. The resistant samples cluster at higher values on the *Y*-axis, indicating that the model identifies distinct spectral patterns in this strain compared to the sensitive one, which appears at lower score values. Similarly, Figure 4B shows the results of the GA-LDA model projected onto the samples in a discriminant axis. This model also shows a clear separation between the classes, with high score values for sensitive strains and lower scores for resistant ones. This emphasizes the high discriminative power of both models in effectively classifying bacterial strains.

**Figure 4:**
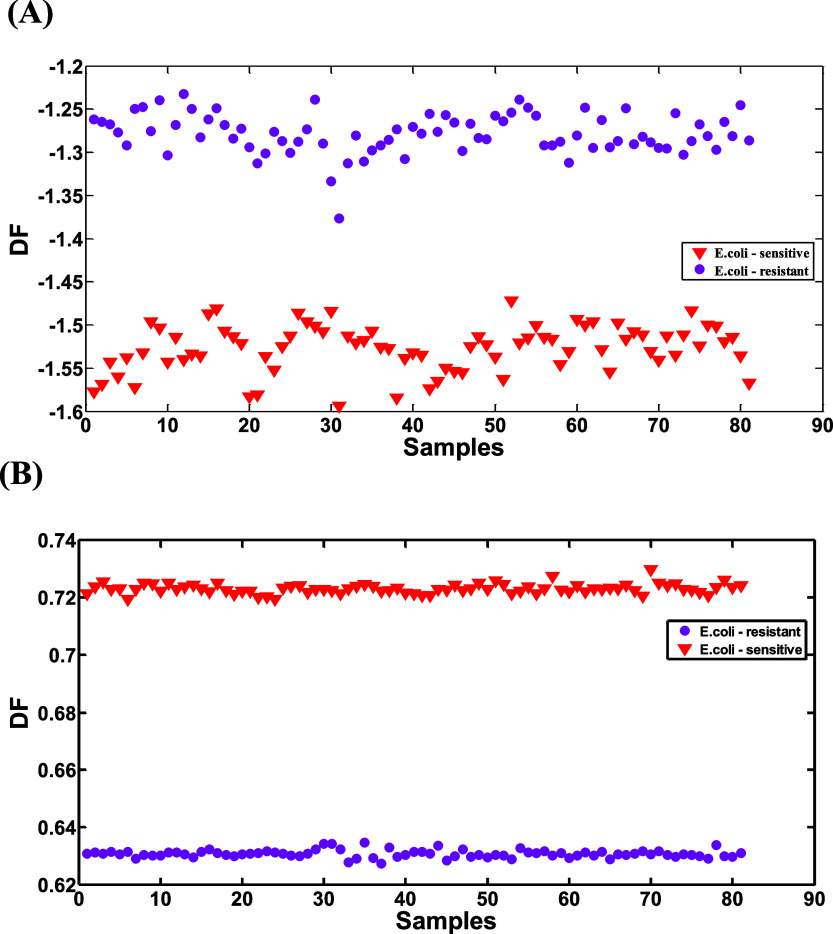
Discriminant functions of SPA-LDA (A) and GA-LDA (B) on NIR spectra for sensitive and resistant *E. coli*.

## 4 Conclusions

This study successfully demonstrated the differentiation between the closely related *E. coli* ATCC (sensitive) and *E. coli* ESBL (resistant) strains using NIR spectroscopy combined with multivariate analysis. Sample preprocessing was performed using the Savitzky–Golay filter with a 15-point window, second-order polynomial, and zero-order derivative, as well as extended multiplicative scatter correction (EMSC).

A total of 162 *E. coli* spectral data points were divided into 114 for training, 24 for validation, and 24 for testing, maintaining a balanced ratio between the two strains—half sensitive and half resistant. The SPA-LDA and GA-LDA models achieved 100% accuracy, sensitivity, and specificity in separating and distinguishing these strains. Both classification models successfully identified the spectral features necessary to robustly demonstrate the differences between the two bacterial strains.

The selected wavelengths correlated satisfactorily with subtle spectral variations in both strains, highlighting the presence of proteins, peptides, nucleic acids, and other metabolic products that constitute the bacterial structure. The proposed method suggests that NIR spectroscopy, combined with multivariate analysis, provides an effective, rapid, and low-cost method for identifying microorganisms such as bacteria, serving as a promising alternative to improve clinical diagnostics and future treatment strategies.
